# Overexpression of *SeTIP2;3* and *SePIP1;4* from *Salicornia europaea* Enhances Drought Tolerance in *Arabidopsis*

**DOI:** 10.3390/ijms27188010

**Published:** 2026-09-09

**Authors:** Ruixin Zhang, Yujie Li, Jiashou Shen, Guangxin Cui, Biao Song, Fang Wu, Lei Xu, Hongshan Yang, Huirong Duan

**Affiliations:** 1 Lanzhou Institute of Husbandry and Pharmaceutical Sciences, Chinese Academy of Agricultural Sciences, Lanzhou 730050, China; 18894019164@163.com (R.Z.); lyj230712@126.com (Y.L.); 19193118123@163.com (J.S.); cuiguangxin@caas.cn (G.C.); s17629810703@163.com (B.S.); wufang@caas.cn (F.W.); xulei@caas.cn (L.X.); 2 Lanzhou Loess Plateau Ecological Environment Key Field Scientific Observation and Experiment Station, Ministry of Agriculture and Rural Affairs, Lanzhou 730000, China

**Keywords:** aquaporins, drought tolerance, *Salicornia europaea*, *SeTIP2;3*, *SePIP1;4*

## Abstract

Aquaporins (AQPs) are key channels for water and small molecule transport across membranes, and represent early targets of stress signaling pathways in plants. *Salicornia europaea* is a typical halophyte adapted to saline-alkaline and nutrient-poor soils. However, studies on AQPs from *S. europaea* and their precise roles in abiotic stress responses remain limited. In this study, *SeTIP2;3* and *SePIP1;4* were cloned from *S. europaea*. Bioinformatics analysis confirmed that they belong to the TIP and PIP subfamilies of AQPs, respectively. Subcellular localization analysis revealed that SeTIP2;3 and SePIP1;4 were localized to the tonoplast and plasma membrane, respectively. Under polyethylene glycol (PEG) treatment, *SeTIP2;3* showed increased transcription levels in the roots, and *SePIP1;4* showed varied degrees of increased transcription in both roots and shoots. Heterologous expression of both genes in *Arabidopsis thaliana* was performed to investigate their functions. Under germination assays, the overexpression (OE) lines exhibited faster germination rates and longer primary roots (1.2–1.4 cm) compared to the wild-type (WT) plants (1.2 cm). Under mannitol-simulated drought stress, the transgenic lines showed reduced MDA content (23.8–28.7 nmol/g), increased accumulation of proline (2.5–3.8 mg/g) and soluble sugars (39.4–49.3 mg/g), and higher chlorophyll content (0.7–0.9 mg/g) than WT plants (30.9 nmol/g; 2.4 mg/g; 30.6 mg/g; 0.7 mg/g). Furthermore, under natural drought conditions, the OE lines displayed lower water loss rates and higher survival rates. Collectively, the findings of this study provide a theoretical basis for the potential application of *Salicornia* aquaporins in enhancing drought tolerance.

## 1. Introduction

Abiotic stresses such as drought, salinity, and temperature extremes disrupt normal physiological, biochemical, and molecular functions in plants, ultimately constraining both vegetative development and reproductive output [1]. To cope with these adverse conditions, plants have evolved a suite of adaptive responses, including enhanced root water acquisition, dynamic adjustments of stomatal aperture, altered biomass partitioning between roots and shoots, accumulation of compatible solutes, and effective detoxification of reactive oxygen species [2,3,4]. Among the promising molecular candidates for bolstering stress tolerance, aquaporins (AQPs) have attracted considerable interest due to their pivotal role in cellular water homeostasis. These evolutionarily conserved integral membrane proteins are situated in both plasma and organellar membranes, where they govern the directional movement of water and various solutes across cellular compartments [5].

In plants, radial water transport in roots occurs through apoplastic, symplastic, and transmembrane pathways [6]. Among them, the transmembrane pathway mediated by AQPs plays an important role, especially in maintaining water and osmotic balance in plant cells [7,8]. Based on sequence homology, AQPs in higher plants are mainly classified into five subfamilies: plasma membrane intrinsic proteins (PIPs), tonoplast intrinsic proteins (TIPs), nodulin 26-like intrinsic proteins (NIPs), small and basic intrinsic proteins (SIPs), and X-intrinsic proteins (XIPs) [9,10]. Among these, PIPs and TIPs represent the two major subfamilies and have been most extensively studied for their roles [11]. Heterologous overexpression of AQP genes has been widely reported to enhance drought tolerance in transgenic plants. For instance, heterologous expression of *FaPIP2;1* from *Festuca arundinacea*, *ScPIP1* from *Simmondsia chinensis*, *MaPIP1;1* from *Musa acuminata*, *GhPIP2;7* from *Gossypium hirsutum*, and *VfPIP1* from *Vicia faba* improved drought resistance in *Arabidopsis* [12,13,14,15,16]. Similarly, upregulation of *PgPIP1.3* and *PgPIP1.4* in *Pennisetum glaucum* coincides with enhanced root hydraulic conductance [17]. In addition, overexpression of *TaTIP41* in transgenic *Triticum aestivum* enhances drought resistance by promoting ABA-induced stomatal closure, increasing proline content, and reducing malondialdehyde (MDA) content [18]. In addition to their involvement in osmotic homeostasis, transgenic overexpression of *OsPIP2.2* in *Oryza sativa* has been shown to promote drought tolerance, with antioxidant modulation serving as an additional contributing mechanism [19]. Collectively, these findings indicate that AQPs participate in plant adaptation to drought stress via diverse and stress-responsive regulatory mechanisms. However, most of these studies have focused on AQPs from glycophytic plants, whereas the functional characteristics of AQPs from halophytes remain limited.

Halophytes, which naturally thrive in high-salt environments, have evolved superior mechanisms to cope with severe osmotic stress [20]. A key feature of stress adaptation in halophytes is their exceptional osmotic adjustment capacity, which enables them to maintain cellular water balance under fluctuating osmotic conditions. Salinity or drought causes rapid shifts in cellular water status, which triggers osmotic imbalances [20]. We therefore reasoned that AQPs from extreme halophytes, which have evolved under persistent osmotic fluctuations, might exhibit transcriptional responsiveness to water deficit.

*Salicornia europaea*, an annual euhalophyte in the Amaranthaceae family, is widely distributed in coastal salt marshes and inland saline-alkaline tidal flats [21,22,23]. It is regarded as a highly promising pioneer crop for saline agriculture and marginal land reclamation [24]. As one of the most salt-tolerant plant species, *S. europaea* maintains water homeostasis through vacuolar Na^+^ sequestration and osmoprotectant accumulation, establishing a favorable water potential gradient that facilitates transmembrane water transport [11,25]. In our previous work, we found that *SeTIP2;3* and *SePIP1;4* exhibited dominant transcript abundances among all *AQP* family members under both control and salt-treated conditions (Unpublished data). Interestingly, their expression was significantly induced by polyethylene glycol (PEG)-simulated drought stress. Given their prominent expression levels and strong inducibility under osmotic stress, we hypothesized that SeTIP2;3 and SePIP1;4 may play important roles in dehydration responses, and that their overexpression in *Arabidopsis* could contribute to improved water retention and osmotic balance under drought conditions.

In this study, we characterized two AQP genes, *SeTIP2;3* and *SePIP1;4*, investigating their basic molecular features through bioinformatic analysis, determining their subcellular localization, and evaluating their physiological effects on drought tolerance, with particular focus on water retention capacity, osmotic adjustment, and oxidative stress mitigation. Our findings provide a theoretical basis for understanding the potential application of *S. europaea* AQPs in improving drought tolerance.

## 2. Results

### 2.1. Cloning and Sequence Analysis of SeTIP2;3 and SePIP1;4

Based on the *S. europaea* genomic database (https://doi.org/10.1093/plphys/kiaf565), we identified the coding sequences (CDS) of *SeTIP2;3* and *SePIP1;4*, corresponding to the gene IDs rna-Seu31234.1 and rna-Seu35638.1, respectively. Using specific primers, we successfully amplified both CDS regions, obtaining fragments of 750 bp and 856 bp, respectively. *SeTIP2;3* encoded a protein of 249 amino acids with a predicted molecular weight of 25.2 kDa. Its theoretical isoelectric point (PI) is 4.7, instability index is 30.7, aliphatic index is 116.4, and grand average of hydropathicity (GRAVY) is 1.0. The *SePIP1;4* CDS encoded a protein of 285 amino acids with a predicted molecular weight of 30.5 kDa. It has a theoretical PI of 8.8, instability index of 29.5, aliphatic index of 94.5, and GRAVY value of 0.3 (Table 1). *SeTIP2;3* and *SePIP1;4* were located on chromosomes 13 and 9, respectively [11].

**Figure 1:**
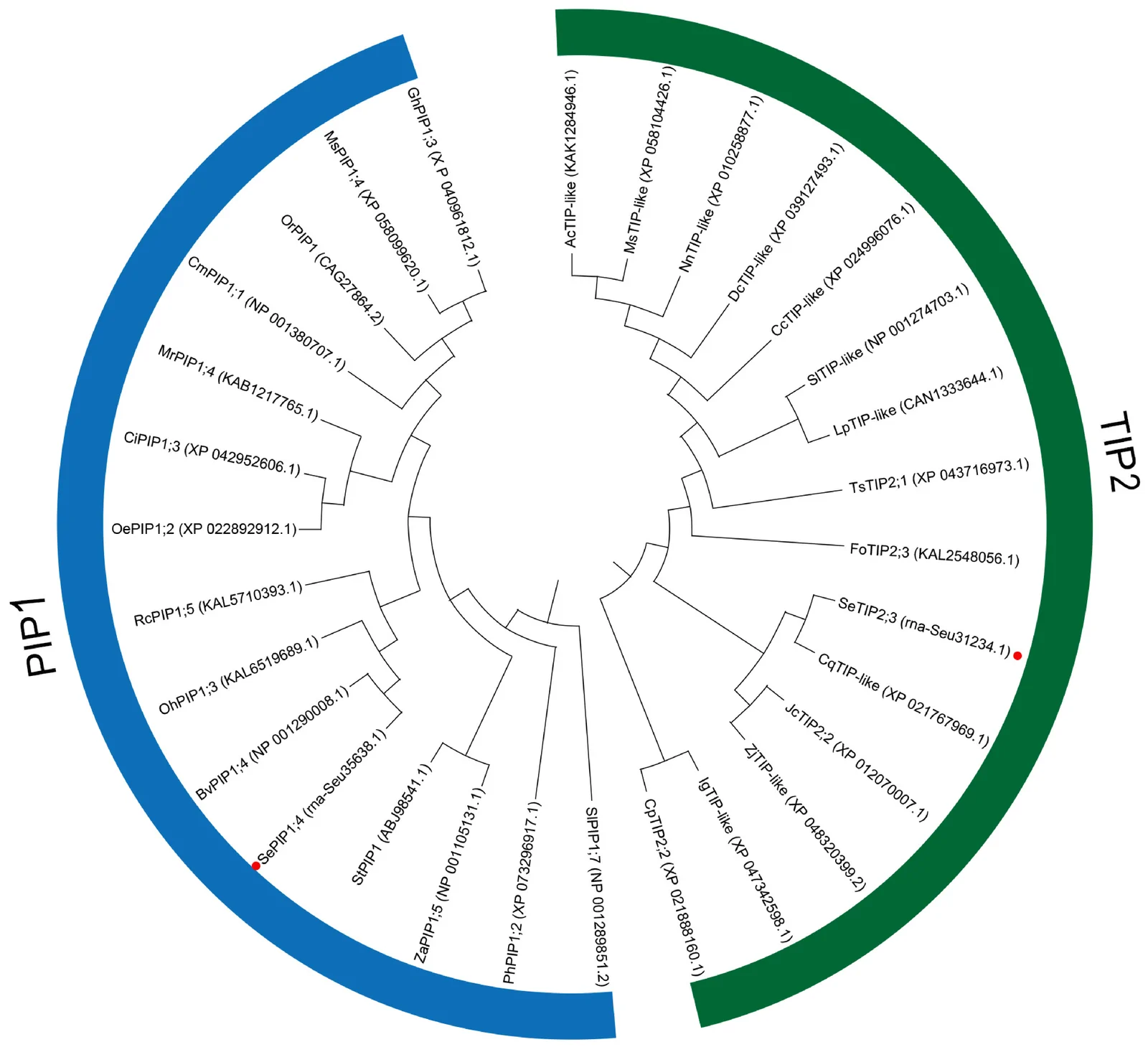
Phylogenetic tree of SeTIP2;3 and SePIP1;4 with TIP and PIPs from other plants. The phylogenetic tree was constructed based on amino acid sequences using the neighbor-joining method in MEGA version 12.0. The gene IDs for *SeTIP2;3* and *SePIP1;4* were obtained from the *S. europaea* genome data, while accession numbers for other homologous genes were sourced from the NCBI database. To enhance clarity, SeTIP2;3 and SePIP1;4 are indicated by red dots.

Phylogenetic analysis revealed that SeTIP2;3 and SePIP1;4 share high homology with TIPs and PIPs from other higher plants, respectively (Figure 1). Specifically, SeTIP2;3 exhibited the highest homology with CqTIP from *Chenopodium quinoa* (88.8%), JcTIP2;2 from *Jatropha curcas* (84.0%), and ZjTIP from *Ziziphus jujuba* (78.4%). Similarly, SePIP1;4 showed high homology with BvPIP1;4 from *Beta vulgaris* (93.0%), OhPIP1;3 from *Orobanche hederae* (82.3%), and OrPIP1 from *Oxybasis rubra* (85.6%).

**Figure 2:**
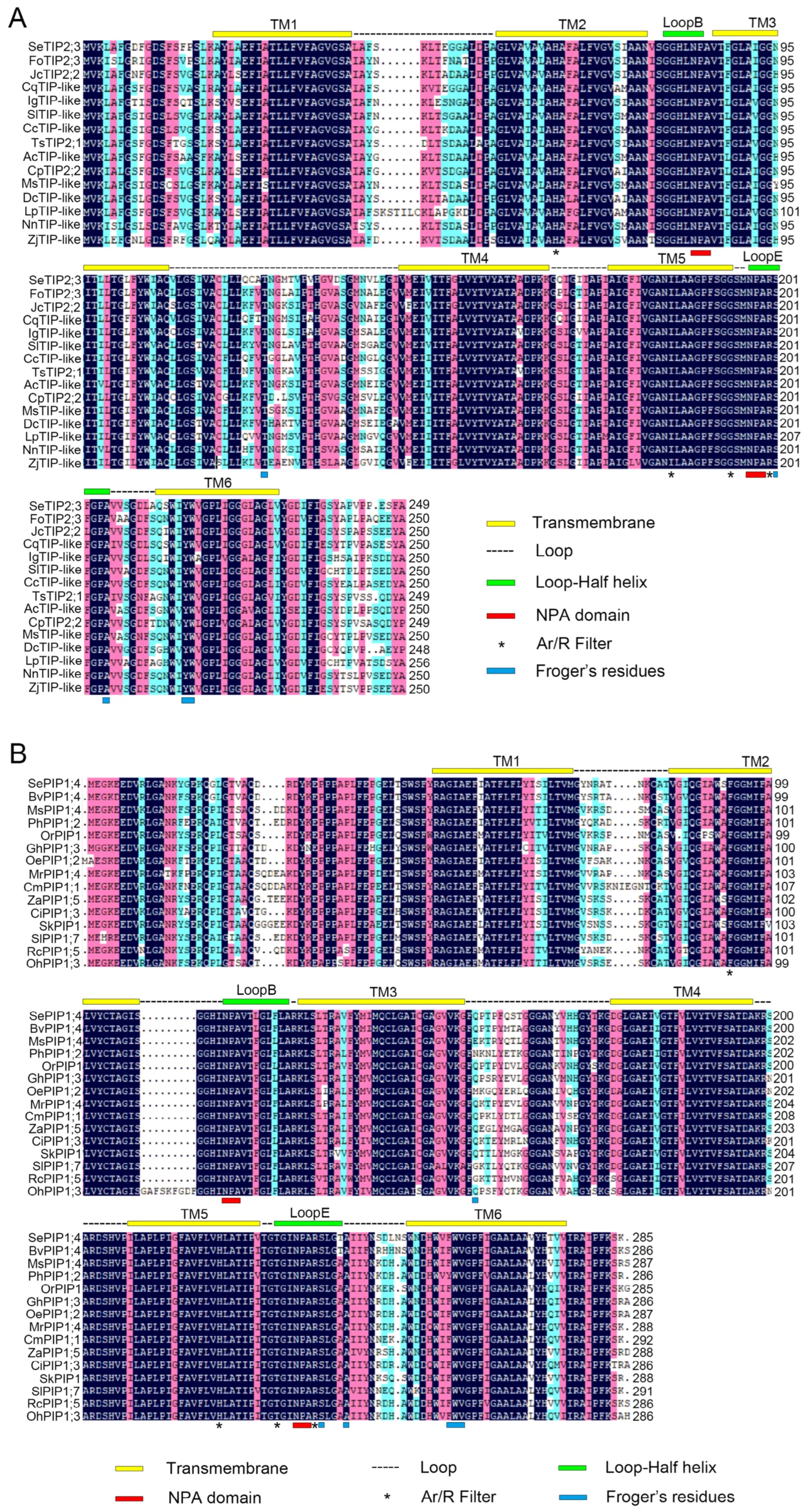
Multiple sequence alignment of SeTIP2;3 (**A**) and SePIP1;4 (**B**) with other plants TIPs and PIPs. The amino acid residues in the alignment are color-coded based on their conservation level. Structural features are annotated as follows: yellow boxes represent the transmembrane helices (TM1-TM6), red boxes indicate the conserved NPA motifs, dashed and green boxes mark loop and half-helix regions, blue boxes denote Froger’s residues, and residues labeled with asterisks (*) correspond to the aromatic/arginine (Ar/R) selectivity filter. The numbers to the right of the amino acids indicate their respective counts.

Sequence analysis indicated that SeTIP2;3 possesses six transmembrane domains, with its N-terminus located extracellularly and its C-terminus intracellularly. In contrast, SePIP1;4 also contains six transmembrane domains, but both its N-terminus and C-terminus are intracellular. Both proteins feature highly conserved dual NPA (Asn-Pro-Ala) motifs, aromatic residue/arginine (ar/R) selectivity filters, and Froger’s residues (Figure 2).

Promoter sequence analysis revealed that the upstream regions of *SeTIP2;3* and *SePIP1;4* harbored multiple cis-acting regulatory elements (CREs) implicated in stress-responsive, hormone-responsive, light-responsive, MYB-related, and development-related processes (Supplementary Figure S1). Among these, STRE elements were particularly abundant in both promoters of *SeTIP2;3* and *SePIP1;4*, while ARE elements were relatively enriched in the *SeTIP2;3* promoter relative to other stress-associated elements.

### 2.2. Drought-Induced Expression of SeTIP2;3 and SePIP1;4 in S. europaea Shoots and Roots

**Figure 3:**
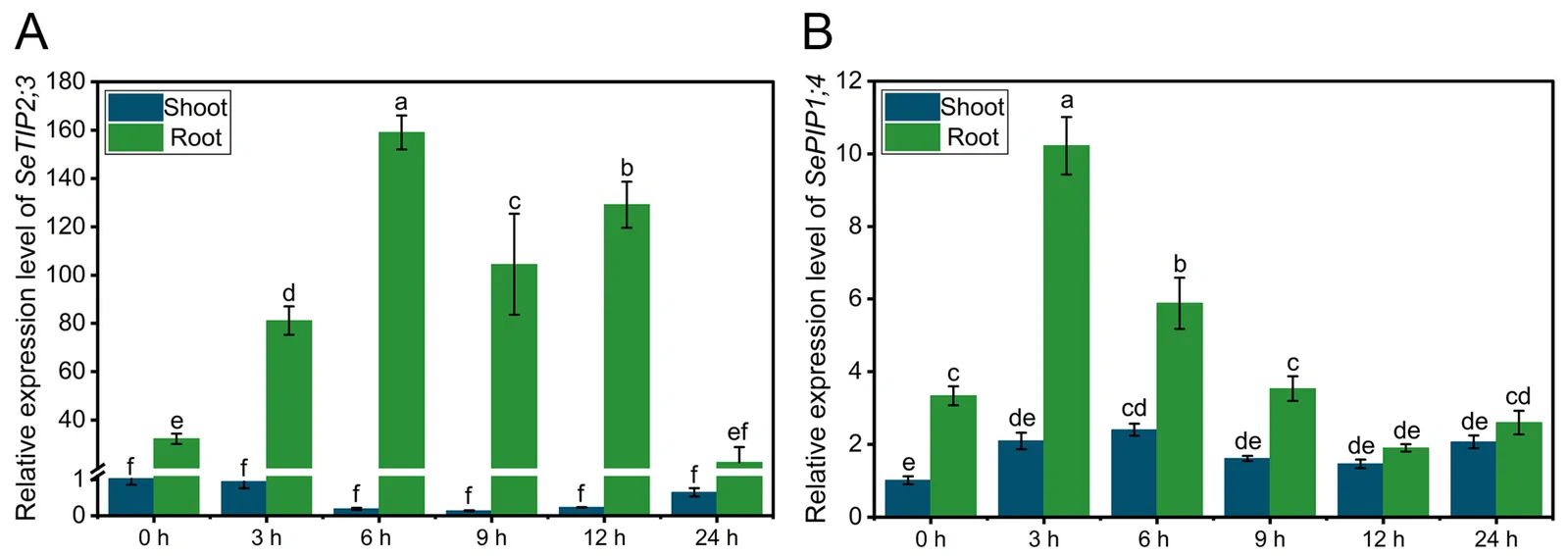
Expression patterns of *SeTIP2;3* (**A**) and *SePIP1;4* (**B**) in shoots and roots of *S. europaea* after treatment with 15% PEG for 0, 3, 6, 9, 12, and 24 h. Different lowercase letters above the bars indicate significant differences among treatments (one-way ANOVA, Duncan’s multiple range test, *p* < 0.05).

To investigate the expression patterns of *SeTIP2;3* and *SePIP1;4* in the shoots and roots of *S. europaea* under PEG-simulated drought stress, five-week-old seedlings were treated with 15% PEG6000 for 0, 3, 6, 9, 12, and 24 h. Analysis by Real-Time Quantitative Reverse Transcription Polymerase Chain Reaction (RT-qPCR) revealed that both genes were expressed in roots and shoots. Under PEG treatment, *SeTIP2;3* transcript levels in shoots showed a slight downregulation over time, although no significant changes were observed compared to the control (Figure 3A). In roots, however, the expression was initially upregulated, reaching the highest level at 6 h of treatment, with an increase of 393.0% relative to the control. Subsequently, expression gradually declined and returned to a level not significantly different from the control by 24 h. Similar to *SeTIP2;3*, *SePIP1;4* expression in roots was also induced, peaked at 3 h with an increase of 206.4% compared to the control, and then gradually decreased. In addition, the expression of *SePIP1;4* in shoots was significantly induced at 3 h and 6 h of treatment (Figure 3B).

### 2.3. Subcellular Localization of SeTIP2;3 and SePIP1;4

**Figure 4:**
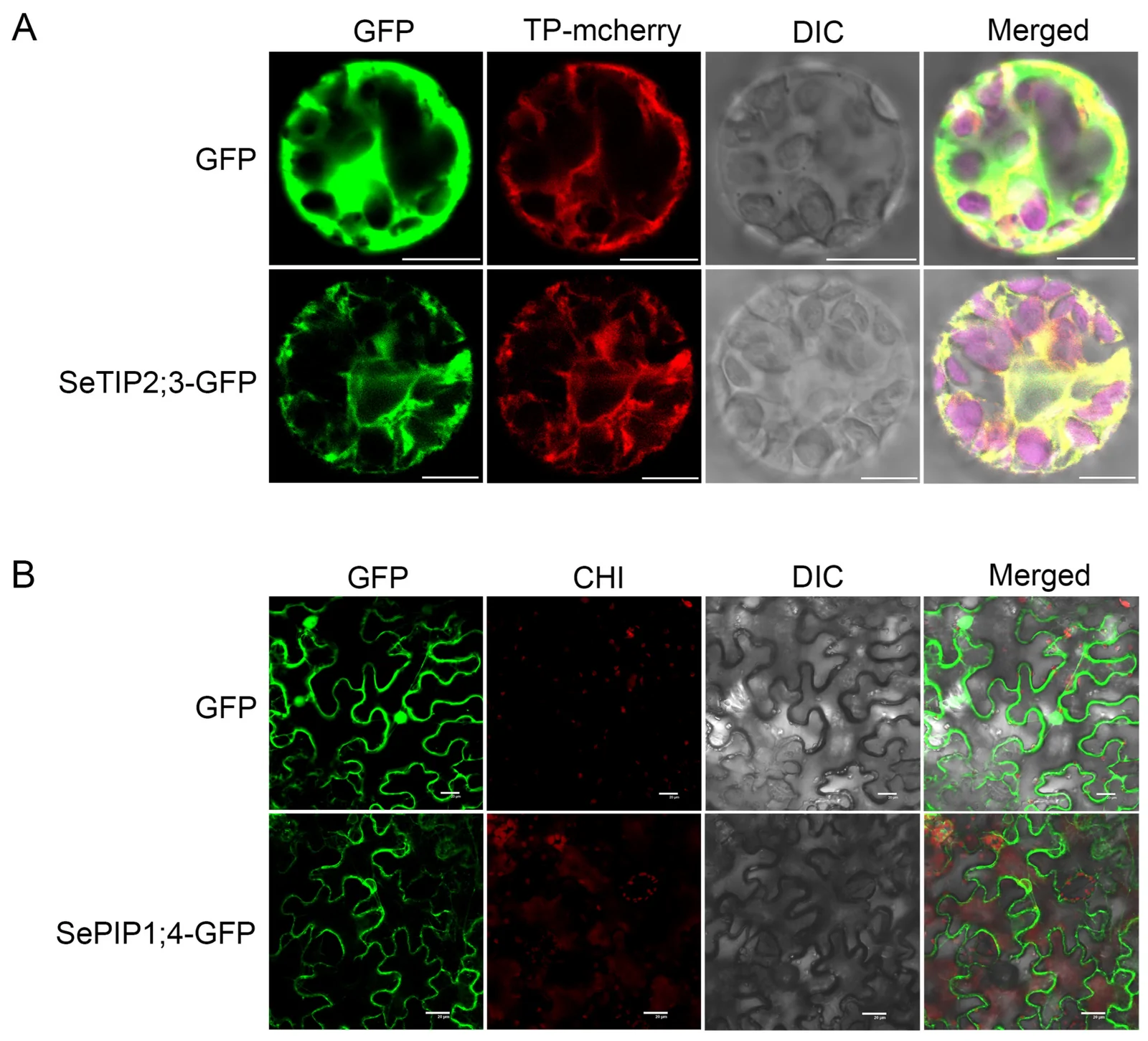
Subcellular localization of *SeTIP2;3* and *SePIP1;4*. (**A**) Subcellular localization of *SeTIP2;3* in *Arabidopsis* protoplasts. The control experiments included expression of GFP via the PBI211 empty vector and expression of the tonoplast marker GFP via *PBI211-35S-GFP-OsTIP2*. Images show: GFP (green fluorescence), TP-mCherry (red fluorescence of the tonoplast marker *OsTIP2*-mCherry), CHI (chlorophyll autofluorescence), DIC (differential interference contrast), and Merge (merged image showing co-localization of GFP and TP-mCherry). Scale bar = 10 μm. (**B**) Subcellular localization of *SePIP1;4* in tobacco cells. The control was GFP expressed via the pCAM empty vector. Images show: GFP (green fluorescent protein), CHI (chlorophyll autofluorescence), DIC (differential interference contrast), and Merge (merged image showing overlay of GFP, CHI, and DIC). Scale bar = 20 μm.

*Arabidopsis* protoplasts were co-transformed with *Agrobacterium tumefaciens* strains GV3101 carrying 35S-SeTIP2;3-GFP and the tonoplast marker OsTIP2-mCherry. The observed co-localization of fluorescence signals at specific sites indicates that SeTIP2;3 is localized to the tonoplast (Figure 4A). After transient expression in tobacco epidermal cells, green fluorescence signals were detected in both the plasma membrane and nucleus for the empty vector, while 35S-SePIP1;4-GFP construct showed fluorescence only on the plasma membrane, suggesting that SePIP1;4 is localized on the plasma membrane (Figure 4B).

### 2.4. Overexpression of SeTIP2;3 and SePIP1;4 Enhances Drought Tolerance in Transgenic Arabidopsis

**Figure 5:**
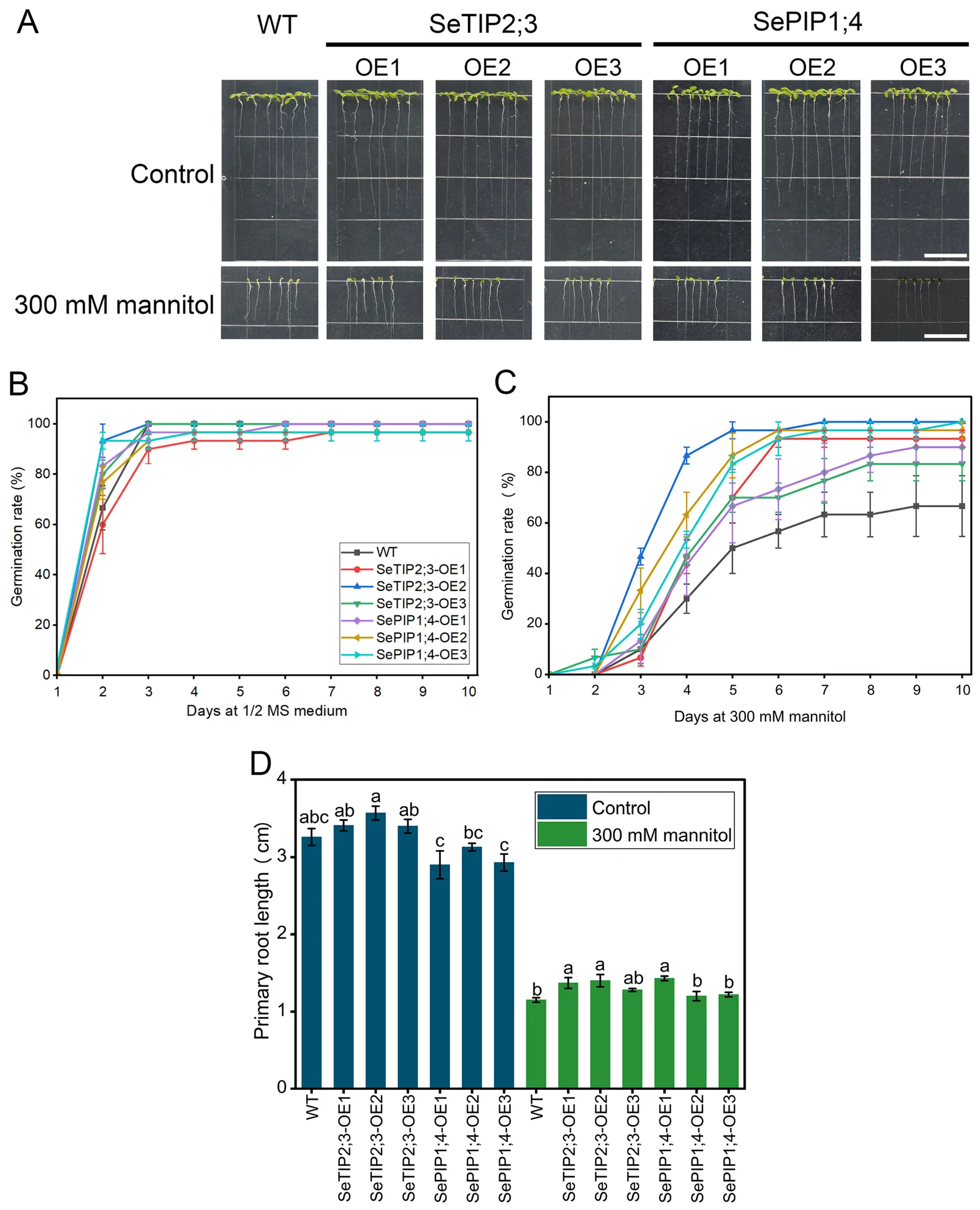
Germination of *Arabidopsis* WT, *SeTIP2;3*-OE, and *SePIP1;4*-OE lines under 300 mM mannitol treatment. (**A**) Phenotypes of *Arabidopsis* WT and OE lines grown for 10 days on 1/2 Murashige and Skoog (MS) medium supplemented with 0 or 300 mM mannitol. (**B**) Germination rates of *Arabidopsis* seeds under control conditions. (**C**) Germination rates of *Arabidopsis* seeds under 300 mM mannitol treatment. Different colors in the figure represent the same lines as in B. (**D**) Primary root length analysis. Data represent means ± SE. Different lowercase letters indicate significant differences under the same treatment (*p* < 0.05).

To investigate the role of *SeTIP2;3* and *SePIP1;4* in drought tolerance, a series of physiological experiments were conducted to compare *Arabidopsis* wild-type (WT) plants with OE lines. First, drought stress during germination was simulated by supplementing the growth medium with 300 mM mannitol (Figure 5). Under normal conditions, the germination rates of WT and OE lines were comparable. However, under 300 mM mannitol treatment, all transgenic lines exhibited significantly higher germination rates compared to WT (Figure 5A–C). Similarly, no significant differences in root length were observed between WT and OE lines under control conditions. In contrast, under 300 mM mannitol-induced stress, the transgenic lines showed increased root lengths relative to WT, particularly in *SeTIP2;3*-OE1, *SeTIP2;3*-OE2, and *SePIP1;4*-OE1 (Figure 5A,D).

**Figure 6:**
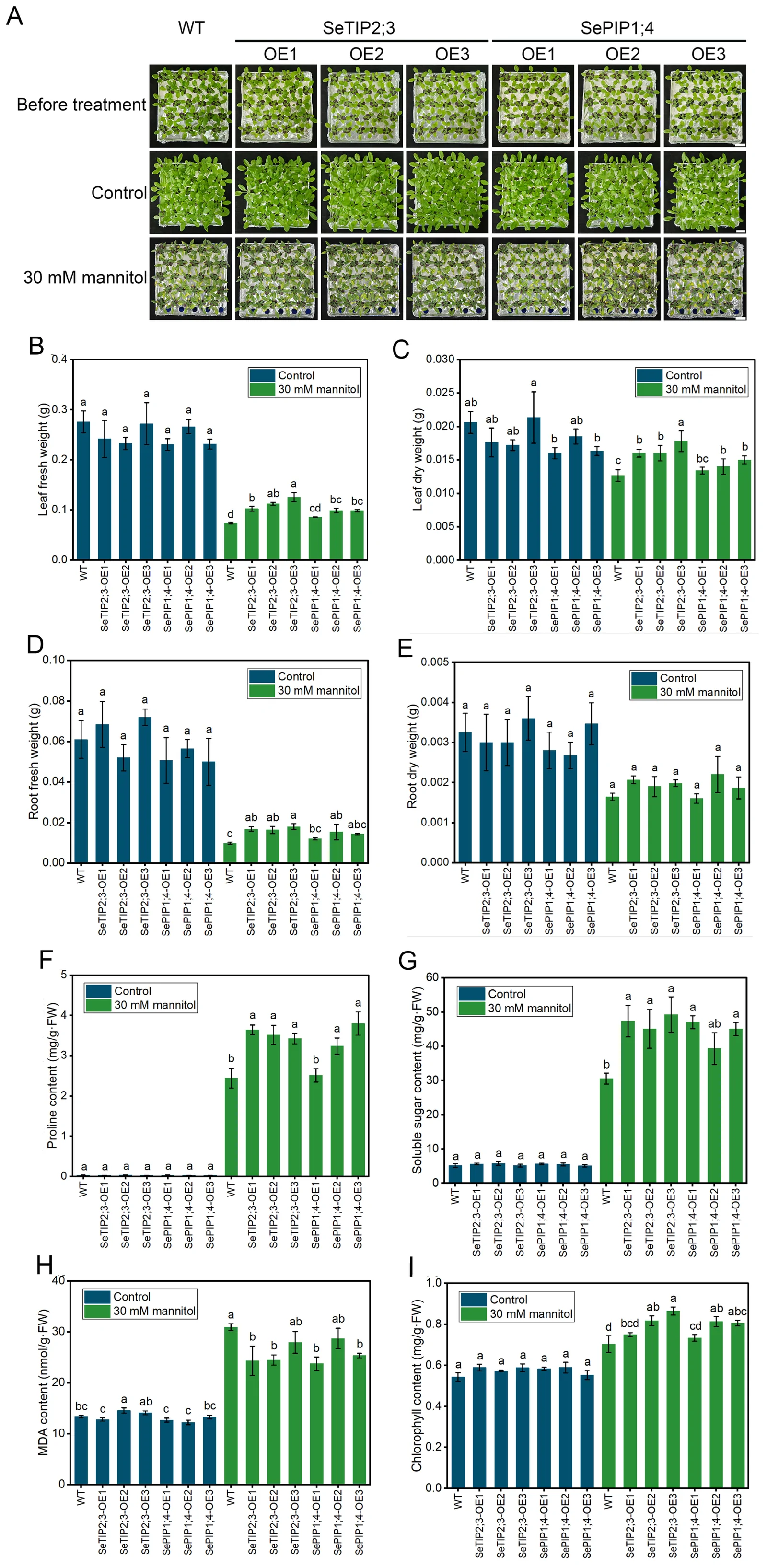
Growth and physiological changes in seedlings of *Arabidopsis* WT, *SeTIP2;3*-OE, and *SePIP1;4*-OE lines under 30 mM mannitol treatment. (**A**) Phenotype of 3-week-old *Arabidopsis* seedlings after 6 days of treatment with 30 mM mannitol. (**B**) Leaf fresh weight after 6 days of 30 mM mannitol treatment. (**C**) Root dry weight after 6 days of 30 mM mannitol treatment. (**D**) Leaf dry weight after 6 days of 30 mM mannitol treatment. (**E**) Root dry weight after 6 days of 30 mM mannitol treatment. (**F**) Proline content. (**G**) Soluble sugar content. (**H**) MDA content. (**I**) Chlorophyll content. Different lowercase letters above the bars indicate significant differences among treatments (one-way ANOVA, Duncan’s multiple range test, *p* < 0.05).

In addition, three-week-old *Arabidopsis* seedlings were treated with 30 mM mannitol. After 6 days of treatment, the fresh and dry weights of the leaves, as well as the fresh root weights, of all transgenic lines were increased to varying degrees compared to WT. Among these, *SeTIP2;3*-OE1, *SeTIP2;3*-OE2, *SeTIP2;3*-OE3, and *SePIP1;4*-OE2 showed particularly significant differences relative to the WT. However, no significant differences in root dry weight were observed between WT and any of the transgenic lines (Figure 6B–E). Physiological analyses revealed that under mannitol treatment, proline content was significantly higher in *SeTIP2;3*-OEs, *SePIP1;4*-OE2, and *SePIP1;4*-OE3 than in WT plants. Soluble sugar content was significantly increased in all transgenic lines except *SePIP1;4*-OE2 compared to the WT. MDA content was significantly lower in *SeTIP2;3*-OE1, *SeTIP2;3*-OE2, *SePIP1;4*-OE1, and *SePIP1;4*-OE3 than in WT. Furthermore, *SeTIP2;3*-OE2, *SeTIP2;3*-OE3, *SePIP1;4*-OE2, and *SePIP1;4*-OE3 exhibited higher chlorophyll content compared to WT (Figure 6F–I).

**Figure 7:**
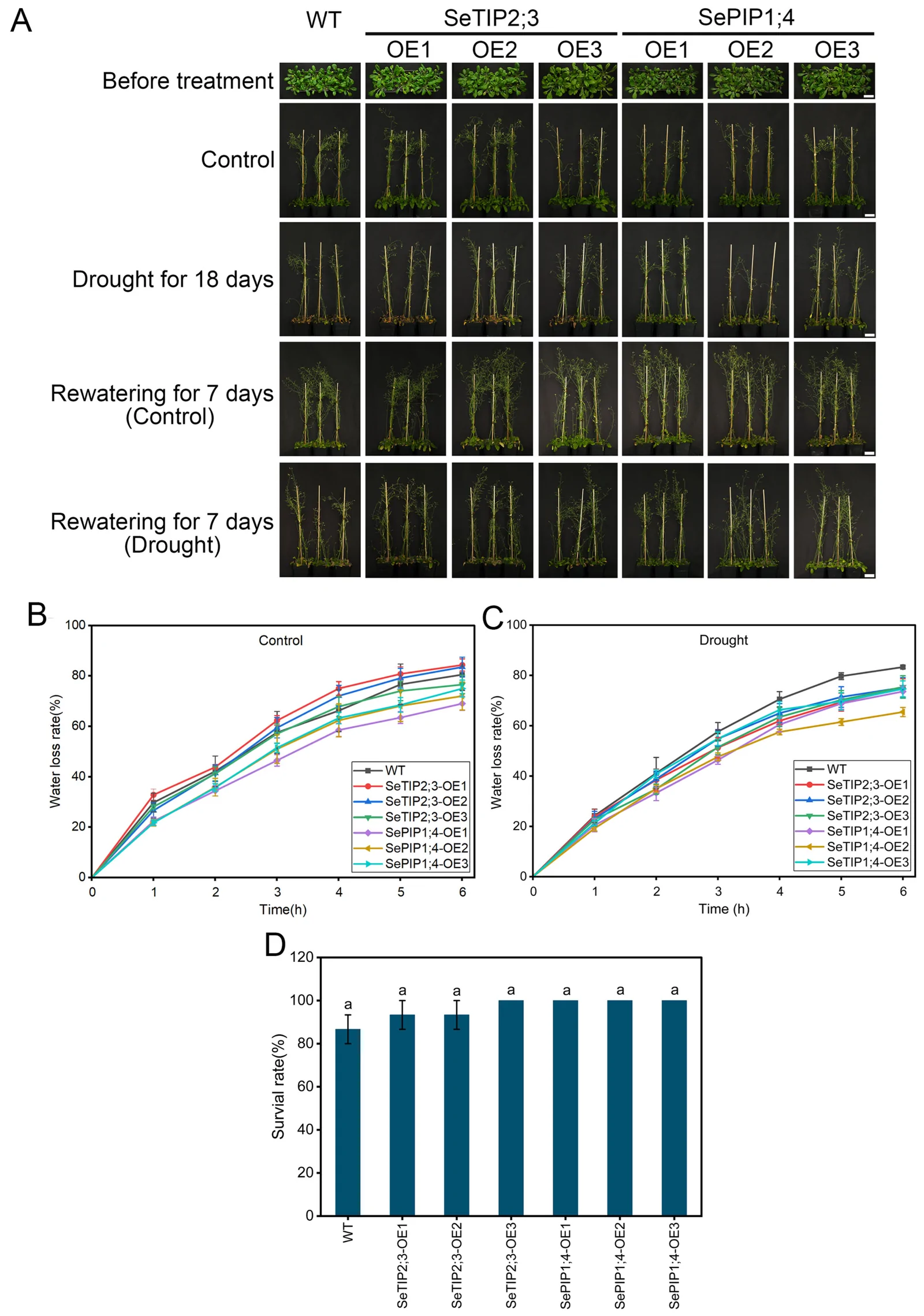
Growth performance, water loss rate, and survival rate of *Arabidopsis* WT, *SeTIP2;3*-OEs, and *SePIP1;4*-OEs seedlings after natural drought treatment of 18 days followed by 7 days of rewatering. (**A**) Phenotype of 3-week-old *Arabidopsis* seedlings. (**B**) Shoot water loss rate under control conditions. (**C**) Shoot water loss rate after natural drought treatment. (**D**) Survival rate statistics. Different lowercase letters above the bars indicate significant differences among treatments (one-way ANOVA, Duncan’s multiple range test, *p* < 0.05).

Finally, three-week-old WT and transgenic lines were subjected to natural drought treatment for 18 days, followed by 7 days of rewatering. Their growth performance was observed, and water loss rates as well as survival rates were recorded (Figure 7). Compared to WT, all transgenic lines exhibited improved growth phenotypes and higher survival rates (Figure 7A,D). In addition, under control conditions, no significant differences in water loss rate were observed among the lines; however, after drought treatment, all transgenic lines showed reduced water loss compared to WT (Figure 7B,C). Collectively, these results indicate that overexpression of *SeTIP2;3* and *SePIP1;4* confers enhanced drought tolerance in *Arabidopsis* to some extent.

## 3. Discussion

### 3.1. SeTIP2;3 and SePIP1;4 Encode Typical Tonoplast and Plasma Membrane AQPs

This study successfully cloned and identified the AQP genes *SeTIP2;3* and *SePIP1;4* from *S. europaea*. Phylogenetic analysis and sequence homology comparisons revealed that the amino acid sequences of *SeTIP2;3* and *SePIP1;4* share high homology with those of other plant species. Both proteins possess six typical transmembrane domains and other conserved motifs (the NPA motif, the Ar/R selectivity filter, and Froger residues) commonly found in most known TIPs and PIPs. The NPA motif and its neighboring amino acid sequences are considered essential for maintaining water transport activity; they force water molecules to reorient as they pass through the channel, thereby preventing proton leakage and maintaining the electrochemical gradient across the membrane [26]. The function of filter and Froger residues is analogous to that of a size-and charge-selective filter; it allows water molecules to pass through while excluding larger or charged solutes [27,28,29]. The above findings strongly support the evolutionary conservation of AQP proteins across the plant kingdom, suggesting that *SeTIP2;3* and *SePIP1;4* may play similar fundamental roles in water transport and homeostasis as their counterparts in other plants. However, direct experimental validation of their water transport activity is still needed in future studies.

### 3.2. Overexpression of SeTIP2;3 and SePIP1;4 Improves Drought Tolerance by Modulating Water Relations and Osmoregulation

Numerous studies have demonstrated that AQP genes are responsive to drought stress [30,31,32]. For instance, expression of *GmPIP2;9* in soybean (*Glycine max*) roots and leaves was significantly upregulated under PEG treatment [33]. The transcript abundance of *HvTIP3;1* and *HvTIP4;1* in barley (*Hordeum vulgare*) leaves increased markedly after drought exposure [34]. Similarly, expression of *OsSIP1* and *OsSIP2* in rice (*O. sativa*) was significantly induced under PEG stress [35]. As previously reported, 15% PEG6000 induces an osmotic potential of about −0.3 MPa, corresponding to moderate water stress. This treatment has been extensively used in studies of drought responses in a variety of species, such as *Cyclocarya paliurus*, soybean, tomato, and so on [36,37,38]. Our study observed that exposure to 15% PEG resulted in significant upregulation of *SeTIP2;3* transcript levels in the roots, and a marked upregulation of *SePIP1;4* expression in both roots and shoots. Concurrently, CREs analysis uncovered several stress-responsive elements, providing additional evidence for the possible roles of these two genes in drought stress responses. We further investigated the drought tolerance capacity of transgenic *Arabidopsis* overexpressing these genes. Germination assays revealed that transgenic lines exhibited higher germination rates and longer primary roots under drought stress; this provides indirect evidence that overexpressing *SeTIP2;3* and *SePIP1;4* enhances drought resistance during the germination stage. Similar observations have been reported by Wang et al. [14] and Patankar et al. [39]. Small molecules of mannitol can enter the solution system more quickly and act directly on the surface of root cell membranes. Therefore, simulated drought stress is an effective way to study drought resistance [40]. Drought stress triggers rapid accumulation of reactive oxygen species (ROS), leading to membrane damage and increased lipid peroxidation [41]. MDA, a product of ROS-induced membrane lipid peroxidation, serves as an indicator of oxidative damage in plants [42]. Overexpression of *AQPs* from various plants has been shown to reduce MDA content under drought stress [1,15]. Correspondingly, our study found that *SeTIP2;3*-OE and *SePIP1;4*-OE lines accumulated lower MDA levels than the WT under drought conditions. Accumulation of osmolytes is considered a crucial mechanism for maintaining osmotic adjustment in plants subjected to drought stress. Osmoprotectants such as proline and soluble sugars help enhance cellular protection under adverse conditions [43]. In this study, overexpression of *SeTIP2;3* and *SePIP1;4* led to increased accumulation of proline and soluble sugars in transgenic *Arabidopsis* under 30 mM mannitol-simulated drought stress compared to the WT. This suggests that *SeTIP2;3* and *SePIP1;4* may contribute to osmotic homeostasis during drought. Furthermore, transgenic lines exhibited higher chlorophyll content than the WT under stress, indicating a potential role in maintaining photosynthetic activity and thereby improving drought tolerance. The ability to maintain water balance is critical for plant adaptation to drought. TIPs and PIPs play essential roles in regulating plant water relations under water deficit conditions [27,28]. We observed that transgenic lines displayed higher survival rates and lower water loss rates under natural drought treatment, suggesting that *SeTIP2;3* and *SePIP1;4* may play a positive role in responding to water scarcity. Collectively, these results demonstrate that *SeTIP2;3* and *SePIP1;4* enhance the drought tolerance of transgenic *Arabidopsis* by mitigating membrane damage, increasing the levels of organic osmolytes and chlorophyll content, thereby improving overall growth performance under drought stress. Therefore, *SeTIP2;3* and *SePIP1;4* are potential candidate genes for crop breeding.

## 4. Materials and Methods

### 4.1. Plant Materials and Treatments

The *S. europaea* seeds used in this study were collected from Jingtai County, Baiyin City, Gansu Province, China. The seeds were sown in sterilized sand and transferred to a growth chamber set at 25/22 °C with a 16 h/8 h (light/dark) photoperiod, and irrigated with modified 1/2 Hoagland nutrient solution. After four weeks of growth, seedlings were transferred to hydroponic boxes and grown for an additional week. Subsequently, the plants were subjected to stress treatment by adding 15% PEG6000 to the nutrient solution. Samples of fresh roots and shoots were collected at 0, 3, 6, 9, 12, and 24 h after treatment, immediately frozen in liquid nitrogen.

The *Arabidopsis* WT used in this study was the Columbia-0 ecotype. Seeds of both WT and transgenic lines were surface-sterilized with 75% ethanol for 5 min, rinsed 5–6 times with sterile deionized water, and then stratified at 4 °C for 3 days. Using 25/22 °C, 16/8 h (light/dark) photoperiod, 60% relative humidity (RH), and 100 μmol m^−2^ s^−1^ Light intensity for growth conditions [44].

For germination assays, seeds were plated on 1/2 MS medium (pH 5.8) containing 0 or 300 mM mannitol plus 1% sucrose (*w*/*v*), solidified with 1% (*w*/*v*) agar [45,46]. Germination was monitored daily for 10 consecutive days, with germination defined by the emergence of a 1 mm radicle from the seed coat. Germination rates and primary root lengths were recorded at the end of the assay. Each treatment included three replicate plates, with 10 seeds per plate.

In hydroponic experiments simulating drought stress with mannitol, three-week-old *Arabidopsis* seedlings of uniform size were treated with either 0 or 30 mM mannitol for 6 days. After treatment, roots were rinsed with deionized water to remove residual mannitol. Root and shoot samples were collected to determine fresh and dry weights, while the remaining shoot tissues were used for physiological assays. Each treatment consisted of at least three independent biological replicates, unless otherwise specified.

For soil-based natural drought experiments, a sterilized mixture of nutrient soil and vermiculite (3:1 ratio by volume) was prepared. Equal amounts (66 g) of this mixture were placed in small pots (the caliber, base diameter, and height are 7 cm, 5 cm, and 8 cm, respectively), with 5 seeds sown per pot in three biological replicates. Three-week-old seedlings were subjected to natural drought stress for 7 days, after which phenotypic photographs were taken, and leaf water loss rates were measured. The remaining seedlings continued under drought conditions for an additional 11 days, followed by a 7-day rewatering period. Survival rates were recorded at the end of the experiment.

### 4.2. Molecular Cloning of SeTIP2;3 and SePIP1;4

Total RNA was extracted from five-week-old *S. europaea* plants using the TaKaRa Mini BEST Plant RNA Extraction Kit (TaKaRa, Kusatsu, Japan, 9769). Subsequently, the extracted RNA was reverse-transcribed into first-strand cDNA with the PrimeScript™ IV 1st strand cDNA Synthesis Kit (with gDNA Eraser) (TaKaRa, Kusatsu, Japan, 6215). The CDS of *SeTIP2;3* were amplified by PCR using PrimeSTAR^®^ Max DNA Polymerase (TaKaRa, Kusatsu, Japan, R045A) with the gene-specific primer pair P1 in Supplementary Table S1. Similarly, the CDS of *SePIP1;4* was amplified using primer pair P2 in Supplementary Table S1.

### 4.3. Bioinformatics Analysis

The basic physicochemical properties of *SeTIP2;3* and *SePIP1;4* were predicted using the online tool TMHMM 2.0 (https://services.healthtech.dtu.dk/services/TMHMM-2.0/, accessed on 13 January 2026) [47]. The basic physicochemical properties of the two proteins were predicted using the ProtParam tool on the ExPASy server (https://web.expasy.org/protparam, accessed on 25 January 2026) [47]. Amino acid sequences of TIPs and PIPs from other plant species, which share high homology with SeTIP2;3 and SePIP1;4, were retrieved from the NCBI database (https://www.ncbi.nlm.nih.gov/, accessed on 6 February 2026). A phylogenetic tree was constructed from these sequences using MEGA software (version 12.0), with 1000 bootstrap replications performed to assess branch support [48]. Multiple sequence alignment of SeTIP2;3 and SePIP1;4 with their homologs was performed with DNAMAN (version 9) software [11]. Promoter regions (2 kb upstream of the start codon) of *SeTIP2;3* and *SePIP1;4* were analyzed for CREs using PlantCARE (http://bioinformatics.psb.ugent.be/webtools/plantcare/html/, accessed on 29 August 2026) [49]. The predicted elements were visualized with TBtools-II (version 2.23+) [49].

### 4.4. RT-qPCR Identification of SeTIP2;3 and SePIP1;4

The kits used for RNA extraction and reverse transcription are the same as above. *SeUBC* from *S. europaea* was used as an internal control. All RT-qPCR experiments were performed with three biological replicates. The relative expression level was calculated using the 2^−ΔΔCt^ method [50]. The RT-qPCR primers used for identifying *SeTIP2;3*, *SePIP1;4* and *SeUBC* were pairs P3, P4 and P5 in Supplementary Table S1, respectively, consistent with those described previously.

### 4.5. Subcellular Localization of SeTIP2;3 and SePIP1;4

Using the NovoRec PCR seamless cloning kit (NovoProtein, Shanghai, China), the CDS of *SeTIP2;3* was amplified with primer pair P6 and inserted into the *Xba*I-digested linearized vector PBI221-EGFP. The resulting recombinant construct, along with the PBI221-EGFP empty vector as a control, was introduced into GV3101. Similarly, the CDS of *SePIP1;4* was amplified with primer pair P7 (Supplementary Table S1) and inserted into the *Xho*I- and *EcoR*I-digested linearized vector pCAM-EGFP. This recombinant construct, together with the empty vector pCAM-EGFP, was introduced into GV3101. These recombinant strains were subsequently used to transform *Arabidopsis* protoplasts and tobacco leaf epidermal cells, respectively.

### 4.6. Overexpression of SeTIP2;3 and SePIP1;4 in Arabidopsis

WT *Arabidopsis* plants were transformed via the floral dip method using the same recombinant strain GV3101 employed for subcellular localization. Positive T_3_-generation transgenic plants for *SeTIP2;3* and *SePIP1;4* were identified by PCR using the primer pairs (P1 for *SeTIP2;3* and P2 for *SePIP1;4*), respectively (Supplementary Figure S2A). Subsequently, three transgenic lines for each gene with relatively consistent expression levels were selected via RT-qPCR, using the primer pairs P3 for *SeTIP2;3* and P4 for *SePIP1;4* (Supplementary Figure S2B,C).

### 4.7. Growth Phenotype and Physiological Analysis

The fresh weight and dry weight of root and shoot were also determined. Proline, soluble sugar, MDA, and chlorophyll contents in *Arabidopsis* were determined using assay kits from Mengxi Biotechnology (Suzhou, China). Additionally, collect similar-sized *Arabidopsis* leaves and immediately weigh them, place them on filter paper, and put them on a clean experimental table (relative humidity of 40%, temperature of 22 °C); there are 4 biological replicates in this experiment. Weigh them every hour for 8 h, and calculate the leaf water loss according to Luo et al. [40].

### 4.8. Data Statistics and Analysis

Data processing and graphing were primarily performed using Origin 2025 software. Statistical analysis was conducted with SPSS 25.0 software, employing one-way analysis of variance (ANOVA) [11]. Duncan’s multiple range test was applied for post hoc comparisons and significance analysis [51]. Figures were assembled using Adobe Photoshop (version 2025).

## 5. Conclusions

The findings of this study confirm the functional roles of *SeTIP2;3* and *SePIP1;4* in drought stress responses. Overexpression of these genes in *Arabidopsis* enhanced drought tolerance by reducing MDA content while increasing the accumulation of proline, soluble sugars, and chlorophyll. These results further elucidate the physiological mechanisms through which the transgenic lines mitigate abiotic stress, indicating that *SeTIP2;3* and *SePIP1;4* may play an active role in reducing membrane damage, maintaining osmotic balance, and improving photosynthetic efficiency.

## Figures and Tables

**Table 1:** Analysis of the basic properties of SeTIP2;3 and SePIP1;4.

Gene Name	Length of CDS (bp)	Number of Amino Acids	Molecular Weight (kDa)	PI	Instability Index	Aliphatic Index	GRAVY
*SeTIP2;3*	750	249	25.2	4.7	30.7	116.4	1.0
*SePIP1;4*	856	285	30.5	8.8	29.5	94.5	0.3

## Data Availability

The original contributions presented in this study are included in the article/Supplementary Materials. Further inquiries can be directed to the corresponding authors.

## Supplementary Materials

The following supporting information can be downloaded at: https://www.mdpi.com/article/10.3390/ijms27188010/s1.
