# Supplementary material for: Overexpression of SeTIP2;3 and SePIP1;4 from Salicornia europaea Enhances Drought Tolerance in Arabidopsis
Source: Int J Mol Sci. 2026 Sep 9;27(18):8010. doi: 10.3390/ijms27188010 (PMC13606676; doi:10.3390/ijms27188010)
Supplement: Supplementary file 1 [file ijms-27-08010-s001.zip › ijms-4521248-supplementary.pdf]

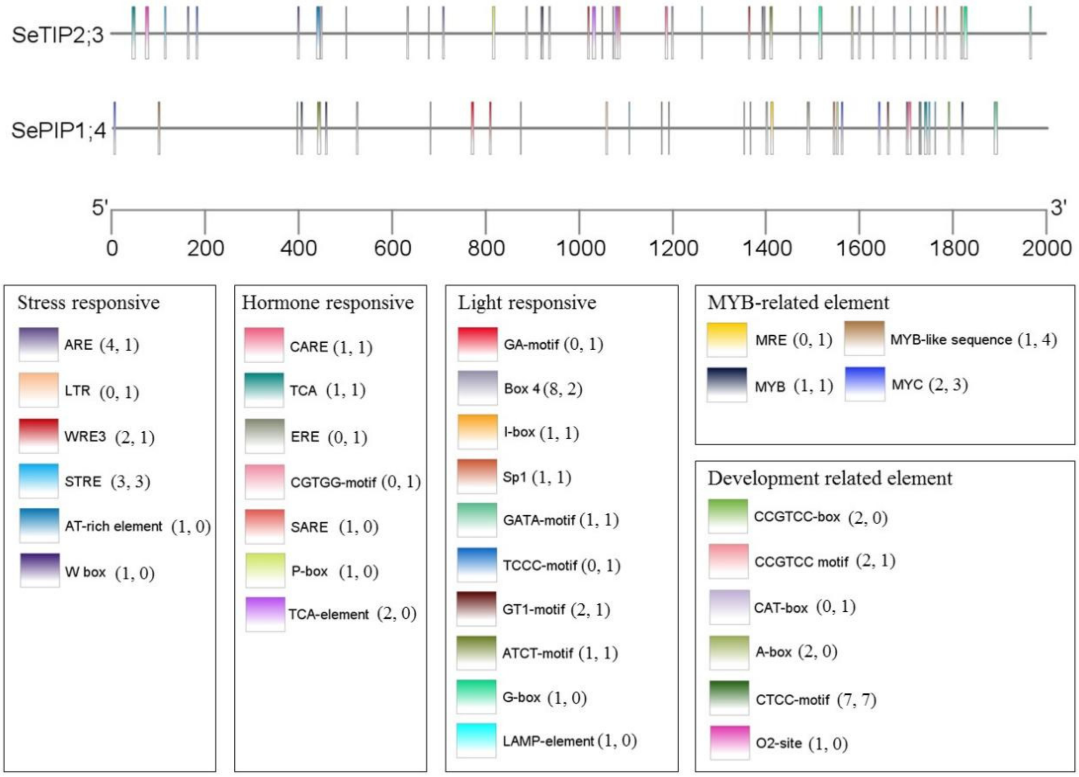

Supplementary Figure S1. Analysis of cis-acting regulatory elements (CREs) in the promoter regions of *SeTIP2;3* and *SePIP1;4* genes. The horizontal gray line represents the 2000 bp promoter sequence upstream of the gene (from 5' to 3'). The short vertical lines in different colors on the horizontal line indicate the relative positions of various predicted CREs. These elements are divided into five functional categories: stress responsive, hormone responsive, light responsive, MYB-related elements, and development-related elements. The numbers in parentheses next to each element name in the legend represent the element number of *SeTIP2;3* and *SePIP1;4*.

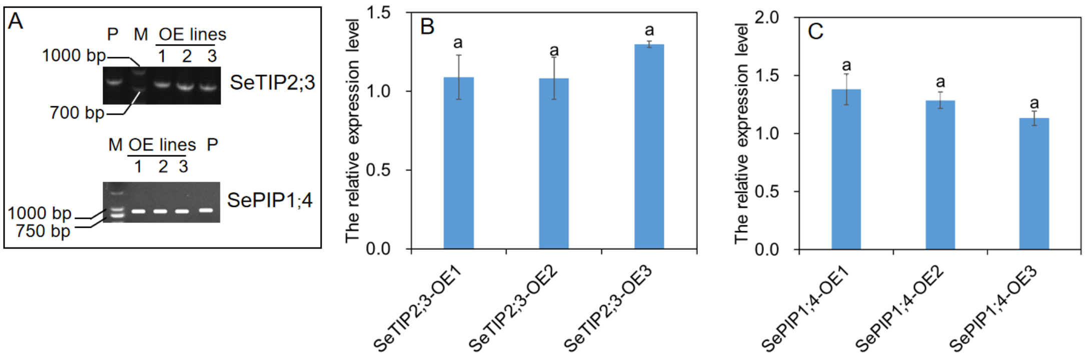

Supplementary Figure S2. PCR and reverse transcription quantitative PCR (RT-qPCR) analysis of target genes in transgenic *Arabidopsis* lines. (**A**) PCR analysis of *Arabidopsis* plants with *SeTIP2;3* and *SePIP1;4* transformation. M: marker; P: plasmid control. (**B**) RT-qPCR analysis of *SeTIP2;3* transformation. (**C**) RT-qPCR analysis of *SePIP1;4* transformation. Values are means ± SE (n = 3) and bars indicate the SE. Duncan's test was used to analyze statistical significance in the same transgenic group, and different letters indicate significant difference ($p < 0.05$).

Supplementary Table S1. Primers used in this article.

Primer Name	F and R	Primer Sequences(5'-3')
P1	F	ATGGTGAAGTTGGCATTTGGTG
	R	AGCAAAACTTTCAGGTGGAACAG
P2	F	ATGGAAGGAAAGGAGGAAGATGT
	R	CTTGGATTTAAATGGGATTGCTCTG
P3	F	GCTACTGCTGCTGACCCTAA
	R	CAGCAAGCCCACCACCAATA
P4	F	ACCCTGCTAGGAGTCTTGGT
	R	ATGCTGCTCCAATGAAGGGT
P5	F	GGGCCAGTCGGTGAAGACAT
	R	ATGCAACCTTTGGGGGCTTGA
P6	F	GAGAACACGGGGGACTATGGTGAAGTTGGCATTTGG
	R	CTCGAATTCGGATCCTCAGCAAAACTTTCAGGTGGAAC
P7	F	TTGGAGAGGACACGCTCGAGATGGAAGGAAAGGAGGAAGATGT
	R	CCCTTGCTCACCATGAATTCCTTGGATTTAAATGGGATTGCTCTG
